# Socioeconomic disparities in diabetes prevalence among the population in Ireland

**DOI:** 10.1186/s12889-025-23022-6

**Published:** 2025-07-02

**Authors:** Gintare Valentelyte, Naomi Holman, Steven James, Nicholas Clarke, Dominika Bhatia, Kathleen Bennett, Jan Sorensen, Edward W. Gregg

**Affiliations:** 1 Centre for Chronic Disease and Population Health Research, School of Population Health, RCSI University of Medicine and Health Sciences, Dublin, Ireland; 2 Department of Epidemiology and Biostatistics, Imperial College London, London, UK; 3 School of Health, University of the Sunshine Coast, Petrie, Australia; 4 Faculty of Medicine, Dentistry and Health Sciences, University of Melbourne, Parkville, VIC Australia; 5 School of Medicine, Western Sydney University, Campbelltown, NSW Australia; 6 Data Science Centre, School of Population Health, RCSI University of Medicine and Health Sciences, Dublin, Ireland; 7 Healthcare Outcome Research Centre, School of Population Health, RCSI University of Medicine and Health Sciences, Dublin, Ireland

**Keywords:** Socioeconomic status, Inequalities, Diabetes prevalence, Deprivation, Ireland

## Abstract

**Introduction:**

A large variation in diabetes prevalence by socioeconomic status (SES) persists internationally. This study aimed to quantify the prevalence of diabetes by age and SES and explore the current levels of inequality in the prevalence of diabetes in Ireland.

**Methods:**

Annual cross-sectional self-reported diabetes data from the national population-based Healthy Ireland Survey for 2015–2023 (*n* = 59,933) were utilised. Highest educational attainment and area-based deprivation were used as SES indicators. Additionally, the differences in diabetes prevalence across population age-groups were reported. Socioeconomic differences and change in inequality over time were quantified using the relative index of inequality (RII). Logistic regression was used to estimate the relative risk (RR) for having self-reported diabetes according to age and SES, adjusted for sex and survey year.

**Results:**

Diabetes prevalence was highest among individuals aged > 75 years (13.1%) compared to those aged < 40 years (1.0%). Similarly, prevalence was highest among the least educated (8.1%; RR = 2.73; 95% CI = 2.38, 3.13) compared to most educated (1.7%) and individuals living in most deprived areas (6.0%; RR = 2.18; 95% CI = 1.76, 2.70) compared to least deprived areas (2.2%). Additionally, the magnitude of relative inequalities as determined by education level were more than twofold greater than the magnitude of inequalities determined by area-based deprivation. Relative inequalities among individuals with diabetes persisted over the period 2015–2023 among the least educated (RII = 3.9; 95% CI = 3.3,4.6) and individuals living in the most deprived areas (RII = 3.65; 95% CI = 2.4,5.5). A slight increase in relative inequalities among the least educated, and a slight decline in relative inequalities among the most deprived was observed, however, these changes over time were not statistically significant.

**Conclusion:**

This is the first study to examine the socioeconomic variation of diabetes prevalence at the Irish population level. Significant differences in diabetes prevalence persist. With the ageing Irish population, this study highlights the need to consider potential effects of diabetes across the older populations and the lowest socioeconomic status groups when implementing equity-oriented diabetes prevention and management programmes.

**Supplementary Information:**

The online version contains supplementary material available at 10.1186/s12889-025-23022-6.

## Introduction

Diabetes has been described as one of the most challenging health problems in the 21st century [1], now affecting more than one in ten adults globally and continuing to increase across all regions of the world [2]. While previous reports demonstrated a significant 3% increase in diabetes prevalence in Ireland from 1998 to 2015, there have been no reports on trends of diabetes prevalence in Ireland for the past 10 years [3]. Since there is no diabetes registry in Ireland, national reports continue to rely on diabetes prevalence estimates from other countries including Scotland [4] and from use of Irish population-level surveys and publicly available sources [5–7]. In 2022, the estimated prevalence of diabetes in Ireland based on Scottish data applied to the Irish population was 6% (*n* = 327,927) [4]. A recent report on the chronic disease burden in Ireland, estimated the prevalence of diabetes in Ireland at 4.6% using Irish population-level survey data [5]. Similarly, in 2017, 164,569 people with diabetes were recorded as eligible for diabetic retinopathy screening in Ireland [6]. However, diabetes prevalence reports in Ireland continue to be limited to specific years, and in some instances, to certain population age groups. Whereas diabetes prevalence continues to grow globally, some high-income countries have observed a peak and plateau in incidence and prevalence rates, particularly among older populations [2, 8].

Socio-economic status (SES), and particularly material deprivation, is a fundamental determinant of diabetes risk [9]. Significant variation in diabetes prevalence by SES has been reported within countries, and increasingly so in low- and middle-income countries [10]. Even where prevalence has peaked, there is generalised concern that the large socio-economic disparities in diabetes risk are either persisting or worsening [9]. The highest prevalence of diabetes have been reported among populations with the lowest level of education [11], those with lowest income, those living in more deprived areas [12, 13] and those who are unemployed [14]. Greater inequalities among the least educated and the most deprived [15] populations continue to persist across countries [10]. Social determinants of health have contributed to these increasing inequalities such as restricted access to nutritious foods and exercise opportunities, lower health literacy, limited healthcare access, and financial barriers [14, 16–19].

Several factors make Ireland a particularly important place to better understand the nature and direction of the relationship between SES and diabetes prevalence. Firstly, Ireland’s changing economic context has led to dynamic economic changes in the European Union [20], including long-term growth, referred to as the Celtic Tiger period [21], followed by the largest proportionate economic decline related to the 2008 recession [22], which was followed by a 10-year recovery [23] until COVID-19. Secondly, Ireland is one of the most frequent destinations for migrants in the European Union [24], which has contributed to changes in the socioeconomic profile and the diversification of the Irish population [25]. Due to migration, the structure of the Irish healthcare system which includes both public and private patients, has been impacted and continues to influence healthcare access and associated disparities among certain population groups [26, 27]. The recent Sláintecare implementation plan to reform the Irish healthcare system towards a universal, single-tier system has been in motion since 2018 to ensure equitable access to healthcare services based on need, including focus across various communities and SES population groups in Ireland [28]. Finally, no prior reports have examined the trends and trajectories of either diabetes prevalence or its relationship to SES in Ireland, and global statistics continue to rely on estimates from 2010 [29].

Our study aimed to provide an updated report on the trends in diabetes prevalence in Ireland and determine the magnitude of SES-related inequalities and whether they are changing. Using the Healthy Ireland Survey, a nationally representative survey of the Irish population, we reported the variation and trends of diabetes prevalence over the period 2015–2023, and explored the presence of potential inequalities in diabetes over this period in Ireland, as determined by age and SES.

## Methods

### Data

We examined population-based cross-sectional data from the *Healthy Ireland* surveys over the period 2015–2023 for the population aged 15 years and older. We included the full sample of individuals in our analysis who had self-reported to have diabetes (yes/no). The Healthy Ireland survey is conducted each year for a representative sample of the population residing in Ireland [30]. Each year, the sample is identified using probability sampling of all electoral divisions around the country, with a random sample of addresses identified from each region using the list of all addresses in the state (GeoDirectory) [31, 32]. The Healthy Ireland survey collects individual data on health, self-reported chronic diseases including diabetes, health behaviours, along with socioeconomic indicators [30, 32]. From 2015 to 2019, the survey was conducted by personal interview. However, due to the COVID-19 pandemic, the survey was not carried out in 2020. From 2021 the survey has been conducted by telephone using random digit dialling [30]. The Healthy Ireland survey has been developed in line with Ireland’s Well-Being Framework to inform and provide research evidence to the Department of Health on the quality of life of the Irish population (access to all surveys: [30]).

This research was approved by the RCSI University of Medicine and Health Sciences ethics committee [REC202401019]. Written and verbal informed consent was obtained from all survey respondents.

#### Socioeconomic status

We defined socioeconomic status using two measures: highest educational attainment and area-based deprivation. Education was captured for each individual in the Healthy Ireland survey and we categorised data into three categories capturing the highest educational attainment from primary to postgraduate level: *Low* - early childhood, primary, lower secondary; *Medium* - upper secondary, post-secondary, short tertiary; *High* - Bachelors, Masters and Doctoral. To capture area-based deprivation, we used the Healthy Ireland survey categorisation. The Healthy Ireland survey uses the Haase Pratschke (HP) deprivation index [33], which is derived from Irish population census data and measures relative deprivation (constructed of demographic profile, social class e.g. lowest and highest educational attainment and labour market affiliation for the local population) across small areas during the period 2016–2019 [30–32]. For the analysis, the index was divided into 5 categories 1 (most deprived) to 5 (least deprived) [31]. During 2015–2023 the classification system capturing deprivation differed in 2015, was only partly captured in 2021, and was not captured from 2022 to 2023 [30]. To maintain measurement consistency, we limited the area-based deprivation measure to the HP index and only included this measure in our analysis over the survey period from 2016 to 2019.

### Analysis

Diabetes prevalence trends and variation for each survey year by age-group and SES were reported descriptively. Additionally, logistic regressions were performed and the estimated odds ratios were converted to estimate the adjusted relative risk ratios (RRs) of self-reported diabetes across the period 2015–2023 combined [34]. The dependent variable was self-reported diabetes (yes/no) and the independent variables were each of the socioeconomic measures (education, area-based deprivation) in separate models. Diabetes prevalence variation was estimated independently for each of the SES indicators, relative to no diabetes. In our estimation, we used the highest level of education and the least deprived category as reference groups. Additionally, in each SES estimation model, we included an interaction between sex and age (< 40, 40–64, 65–74, > 75 year age bands), to adjust for potential differences in self-reported diabetes across these groups. To check the robustness of our estimates for education, we conducted sensitivity analysis by excluding all individuals aged < 30 years i.e. individuals who may not have completed their formal education/training. Additionally, we estimated in a separate logistic model, the age-group differences, using the youngest age-group category (< 40 years) as the reference group, adjusted for sex. Similarly, to capture potential differences across each survey year and to improve accuracy of estimates, we adjusted for survey year. All analyses applied the population sampling weights included in the Healthy Ireland surveys to allow generalisation of the estimates at the Irish population level.

#### Relative index of inequality

In addition to reporting and measuring the variation in diabetes prevalence by age-group and across each SES group using adjusted RR, we explored the extent to which this variation changed during the period 2015–2023. This was done by measuring the socioeconomic inequalities among those with diabetes, using the relative index of inequality (RII). The RII measures and quantifies the proportionate increase or decrease in inequality between individuals in the lowest socioeconomic group relative to the highest socioeconomic group (i.e. relative inequality) by fitting a model to the complete data [35, 36]. This measure can be interpreted as the prevalence of diabetes at the bottom of the educational hierarchy compared to diabetes prevalence at the top of the educational hierarchy [37]. A similar interpretation for area-based deprivation applies. A RII value of one indicates no presence of inequality, and higher values (above one) indicate greater inequality (for individuals in the lowest SES group) [36, 37].

We estimated the RII (using the “siilogit” Stata command [38]), for each individual survey year, and for the entire period (2015–2019 and 2021–2023) by education and area-based deprivation. First, the weighted sample of all individuals was ranked from the most-disadvantaged group e.g. most deprived, to the most-advantaged group e.g. least deprived. Using this approach, we estimated the RII among individuals with diabetes relative to individuals without diabetes. We captured the RII for education over the period 2015–2023, and for area-based deprivation over the period 2016–2019, adjusting for differences in the age profile across each estimation period. These indices were compared between those with and without diabetes.

Finally, we tested for trends in diabetes prevalence over the survey period, by age-group and SES, by conducting a temporal trend test [39]. We did this by estimating separate logistic regressions, with diabetes as the dependent variable, age-group, education and area-based deprivation as independent variables. In each model, we adjusted for sex, age (for education and area-based deprivation) and included survey year as a continuous variable [40]. We additionally applied the population survey weights included in the Healthy Ireland survey. Statistically significant trends were identified based on the significance of the survey year coefficient. This test allowed us to identify whether the trend in diabetes prevalence and the relative inequalities across SES groups were statistically significant over the survey period. Confidence intervals were calculated at the 95% level. All estimations were performed using Stata v.18.0 software [41]. Statistical significance was assumed at *p* < 0.05.

## Results

### Descriptive statistics

**Fig. 1:**
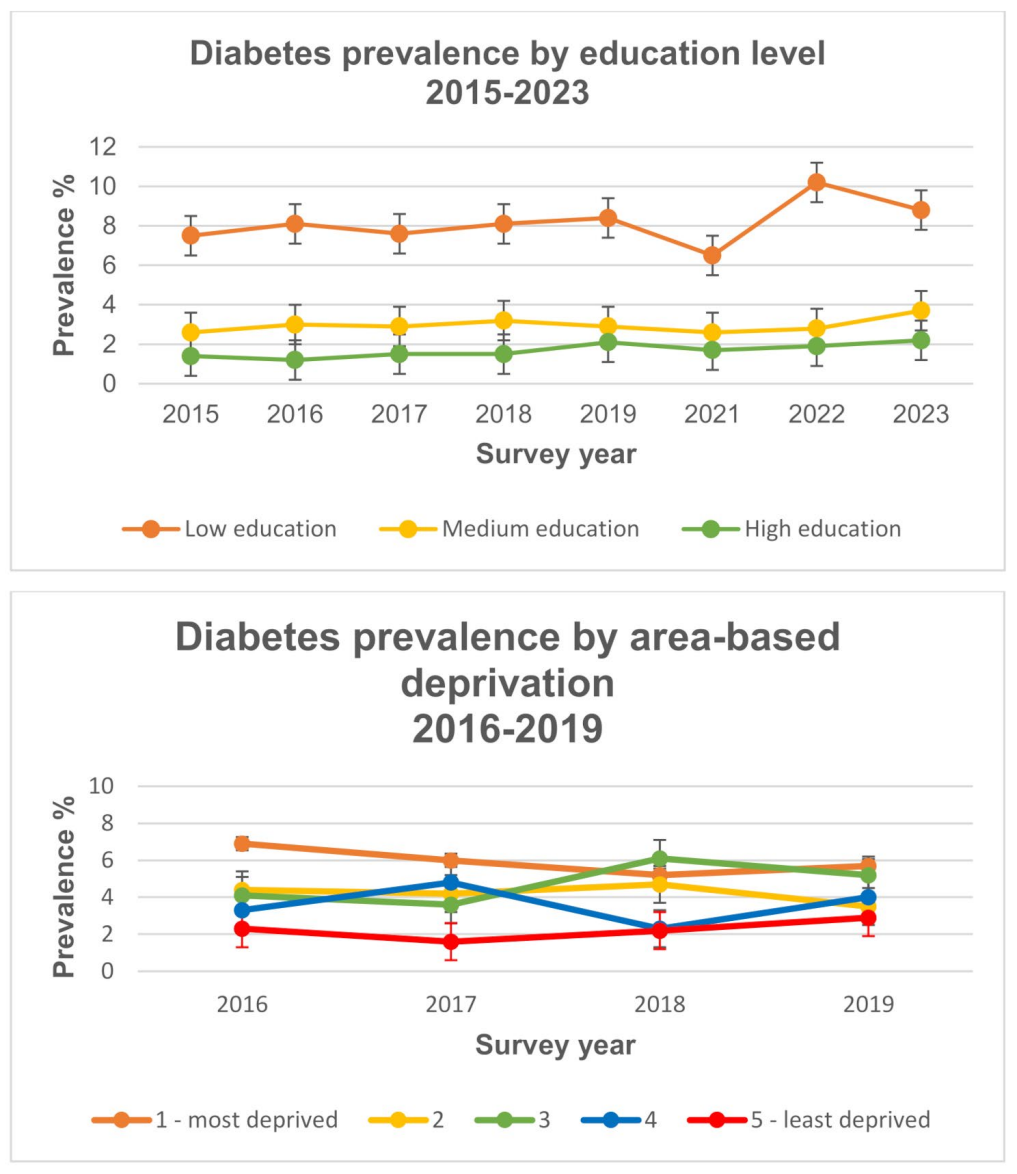
Self-reported diabetes by SES as per Healthy Ireland survey: education (2015–2023) and area-based deprivation (2016–2019). Note: Complete area-based deprivation data was available for years 2016 to 2019 only. Reported prevalence is based on population sampling weights provided in the Healthy Ireland survey

Self-reported diabetes prevalence for 59,933 individuals over the full survey period 2015–2023 is summarised in Table 1 by sociodemographic characteristics (full sample analysed is summarised in Table A5, Supplementary File 6). Over the entire survey period, diabetes prevalence was 4.2%, and ranged from 3.6% to 4.8% (Figure A1, Supplementary File 2). Table A1, Supplementary File 1 provides a summary of diabetes prevalence by individual survey year for each SES indicator and individual demographics. The prevalence of diabetes did not change substantially over this period. Overall, diabetes prevalence was higher for males (5%) and among those aged 75 years and older (13.2%) (Table 1). Further, diabetes prevalence was greater among those with a low level of education (8.1%), compared to those with high level of education (1.7%). Similarly, diabetes prevalence was greatest among individuals in the most deprived group at 6.2%, compared to 2.2% in the least deprived group. Figure 1 illustrates the variation of diabetes by SES for each survey year (Age-group variation is reported in Figure A2, Supplementary File 2). Prevalence remained highest in the oldest age-groups and in the lower educational groups across all survey years (Figure A2, Supplementary File 2). Differences in diabetes prevalence over the survey period were greatest among the least educated relative to the most educated groups. Similar differences, although smaller in magnitude, were observed across area-based deprivation groups, with highest prevalence in diabetes observed among the most deprived groups compared to least deprived.

**Table 1:** Summary of diabetes prevalence by demographic and socioeconomic variables (Healthy Ireland survey 2015–2023)

Diabetes	No	Yes	Total
n (%)	57,390 (95.8)	2543 (4.2)	59,933
**Sex**,** n (%)**			
Male	27,909 (95.0)	1467 (5.0)	29,376
Female	29,473 (96.5)	1075 (3.5)	30,548
**Age group**,** n (%)**			
< 40	24,895 (99.0)	241 (1.0)	25,136
40–64	23,251 (95.5)	1083 (4.5)	24,333
65–74	5654 (89.4)	671 (10.6)	6325
> 75	3589 (86.8)	548 (13.2)	4137
**Education level**,** n (%)**			
Low: </primary, lower secondary	17,271 (91.9)	1525 (8.1)	18,796
Medium: secondary, post-secondary, non-tertiary	24,125 (97.1)	736 (2.9)	24,861
High: 3rd level, BA MA, PhD	15,927 (98.3)	280 (1.7)	16,207
**Deprivation**,** n (%)**			
1 – Most deprived	6098 (94.0)	387 (6.0)	6486
2	5988 (95.8)	263 (4.2)	6251
3	5844 (95.2)	296 (4.8)	6139
4	5804 (96.4)	218 (3.6)	6022
5 – Least deprived	4980 (97.8)	114 (2.2)	5094
**Year**,** n (%)**			
2015	7239 (96.0)	300 (4.0)	7539
2016	7173 (95.7)	325 (4.3)	7498
2017	7174 (95.8)	313 (4.2)	7487
2018	7376 (95.8)	325 (4.2)	7701
2019	7065 (95.6)	323 (4.4)	7382
2021	7189 (96.4)	265 (3.6)	7454
2022	7098 (95.2)	357 (4.8)	7455
2023	7075 (95.5)	336 (4.5)	7411

Note: Reported prevalence is based on population sampling weights provided in the Healthy Ireland survey

### Logistic regression estimates

Table 2 summarises the estimated adjusted RRs by education and area-based deprivation (age-group RRs are summarised in Table A2, Supplementary File 3). Statistically significant differences in diabetes prevalence by age-group were observed. Individuals aged > 75 years had a RR of 10.9 (95% CI: 9.34,12.92) of having diabetes, compared to the youngest age-group (< 40 years). Similarly, statistically significant differences in diabetes prevalence were observed by SES. Individuals with a low level of education had a RR of 2.73 (95% CI: 2.38, 3.13) of having diabetes, compared to those with a high level of education. Our sensitivity analysis for education confirmed the robustness of these estimates (Table A6, Supplementary File 7). Similarly, the RR for the most deprived individuals was 2.18 (95% CI: 1.76, 2.70) compared to least deprived (group 5).

**Table 2:** Adjusted relative risk ratios of self-reported diabetes by SES (2015–2023)

	RR	95% Confidence Interval	
	Education level		
Low	2.73	2.38	3.13
Medium	1.56	1.35	1.79
High	Ref	Ref	Ref
N	59,768		

	Area-based deprivation		
1 – most deprived	2.18	1.76	2.70
2	1.47	1.17	1.84
3	1.77	1.41	2.21
4	1.36	1.08	1.72
5 – least deprived	Ref	Ref	Ref
N	29,984		

Note: Separate logistic regression models were conducted for education (adjusted for sex, age, survey year), area-based deprivation (adjusted for sex, age, survey year). High education, area-based deprivation group 5 were the reference categories. Area-based deprivation was estimated for year 2016–2019 only. Population sampling weights provided in the Healthy Ireland survey were applied in estimation

*: *p* < 0.1; **: *p* < 0.05; ***: *p* < 0.01

### Relative inequalities

**Fig. 2:**
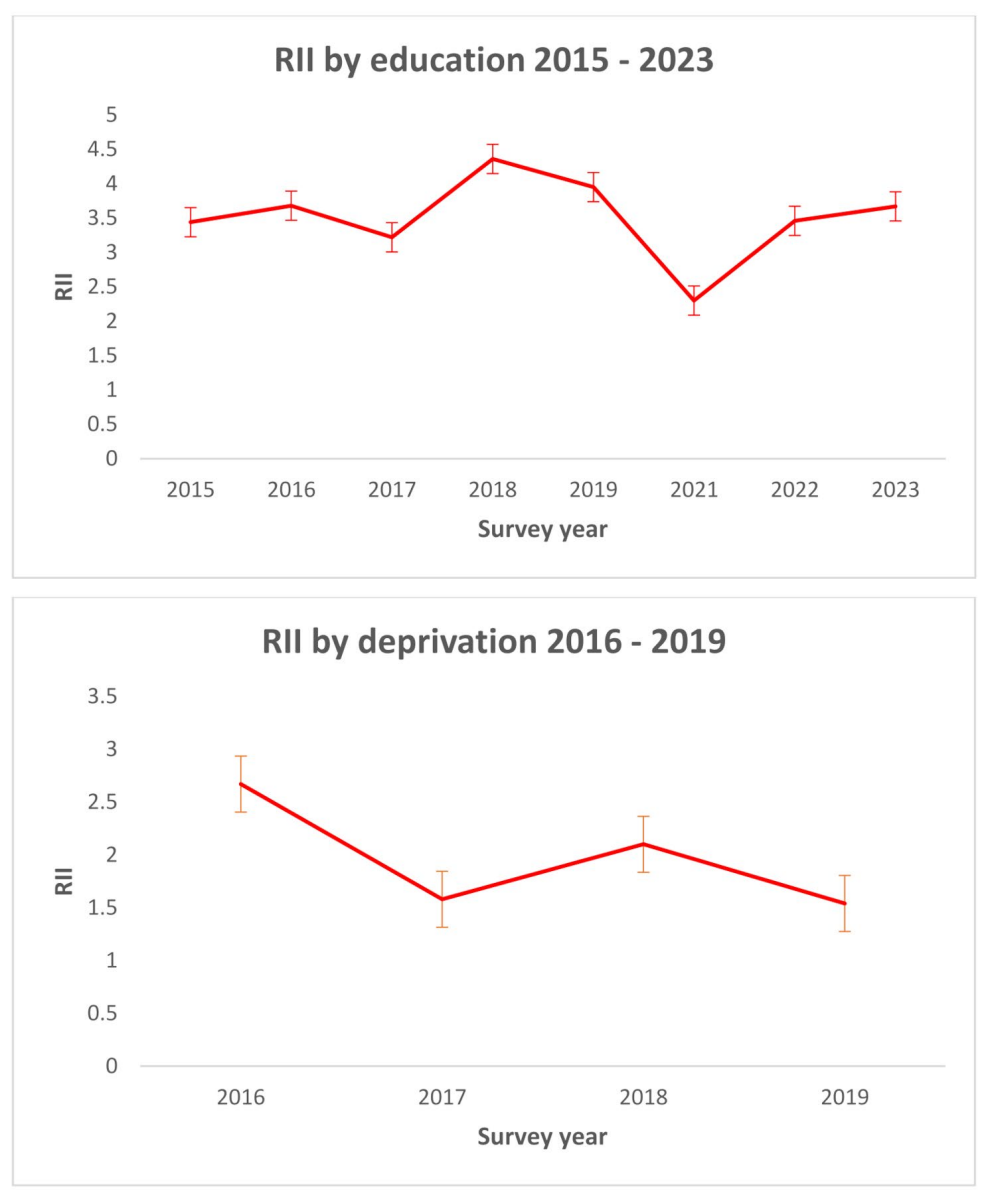
Relative index of inequality (RII) (and 95% CI) by education (2015–2023) and area-based deprivation (2016–2019). Note: adjusted for sex and age. Population sampling weights provided in the Healthy Ireland survey were applied in estimation

The RII estimates (Table A3, Supplementary File 4) suggest significant presence of relative inequalities by SES. The relative inequality by education varied by survey year, and increased slightly, from RII = 3.44 in 2015 to RII = 3.67 in 2023. Diabetes prevalence was persistently higher among those with low education i.e. relative inequalities were more persistent among the least educated individuals with diabetes over the entire survey period (RII = 3.9; 95% CI = 3.31, 4.59) and across each survey year (Fig. 2).

Similarly, we observe significant relative inequality by area-based deprivation, with age-adjusted diabetes prevalence persistently higher among individuals living in the most deprived areas over the survey period 2016–2019 (RII = 3.65; 95% CI = 2.48, 5.37). Over this period, relative inequality in diabetes prevalence across deprivation groups reduced from RII = 2.67 in 2016 to RII = 1.54 in 2019 (Table A3, Supplementary File 4 and Fig. 2). Across each survey year, the magnitude of relative inequalities as determined by education level were more than twofold greater than the magnitude of inequalities determined by area-based deprivation (Table A3, Supplementary File 4).

The linear temporal test of trends over the survey period indicated that the differences in diabetes prevalence (and relative inequalities among the least educated and most deprived individuals with diabetes) across age-groups, education and area-based deprivation were not statistically significant (Table A4, Supplementary File 5). This suggests that these changes over time in relative inequalities were not significant.

## Discussion

Our study provides the most current trends in diabetes prevalence in Ireland and determine the magnitude of SES-related inequalities and whether they are changing. Findings identified significant differences in diabetes prevalence by age and socioeconomic status (SES) groups in Ireland and no apparent reductions in either diabetes prevalence or levels of socio-economic disparities. Diabetes prevalence among the least educated sub-groups (8.1%) is more than four times that of the highest educated groups (1.7%). Similarly, diabetes prevalence among individuals living in the most deprived areas (6%) continued to be highest, three times that of individuals living in the most affluent areas (2.2%). We also observed a steep association of age with diabetes prevalence, as those aged > 75 (13%) have more than 10 times that of persons aged < 40 years (1%). This study provides the first in-depth population-level estimates for Ireland since those reported for 2010, giving diabetes prevalence over the period 2015–2023. It also provides the first in-depth examination of the relationship of socio-economic factors and diabetes prevalence in Ireland. Our results suggest that SES is strongly associated with diabetes prevalence across population sub-groups in Ireland defined by education level and area-based deprivation.

Our findings suggest that Ireland has one of the lower national prevalence estimates in the European Union with the average prevalence of 6.2%, but has relatively high differences according to socio-economic status [42]. Despite the low prevalence of diabetes in Ireland, our prevalence estimates of 4.2% are similar to previous reports on diabetes prevalence in Ireland. For example, diabetes prevalence for the population aged > 18 years in 2015 was reported at 5.2% [3], and in 2019 the reported prevalence was 4.6% [5]. Reports from The Irish Longitudinal Study on Ageing (TILDA), a representative cohort study in Ireland, observed estimates similar to ours, and indicated prevalence among the older groups aged 65–74 (10.6%) and > 75 years (13.2%) and 8.6% among the overall population aged > 50 years [43]. As our estimates represent self-reported diagnosed diabetes, they do not include the additional proportion of the population who have unknown diabetes. Reports from the European Union have reported that about 25% of those are undiagnosed but that this proportion may vary considerably by country [44]. In TILDA, the proportion undiagnosed for the cohort aged > 50 years was quite low (0.9 out of 9.5%, or about 10%), but this proportion may be higher in the general population [43]. However, much of this variation by age could be related to the age structure of the Irish population, with the Irish population reported to be relatively younger than most European Union countries [45]. As the Irish population continues to age, particularly Ireland’s largest cohort born in 1980 [46], we should expect a peak in diabetes prevalence over the next 10–15 years, reaching diabetes prevalence closer to the EU average.

Our findings contribute to existing evidence highlighting significant variation of diabetes prevalence across various socioeconomic groups in other countries. Across 10 European countries, the highest diabetes prevalence was reported across the least educated population groups, ranging from 2.5 to 8.5% [9]. Similarly, in Italy, diabetes prevalence was significantly higher among men and women aged 74 years and older with the lowest level of education, 17.8%% and 14.4%, respectively [47]. In England, individuals living in the most deprived areas had the highest diabetes prevalence (type 1 and type 2) ranging between 17% and 19% [48]. This suggests that other countries face similar differences in diabetes prevalence across SES groups, to Ireland.

Significant inequalities persisted with no clear evidence of narrowing over the survey period with those in the least educated and the most deprived sub-groups experiencing higher prevalence of diabetes. Our results are in line with previous studies which have reported the presence of inequalities among most deprived populations living with diabetes e.g. in the United States (defined by income level) [49]. Relative inequalities among low educated individuals with diabetes in Switzerland, continued to persist, increasing from RII = 1.51 to RII = 2.53, over the period 2005–2017 [50]. In Denmark, inequalities among individuals with type 2 diabetes living in the most disadvantaged municipalities were also reported (inequality index = 1.23) [15]. Similarly, an increase in relative inequalities among individuals with diabetes in England persisted over the period 2005–2012, RII = 1.90 [51].

The fact that socioeconomic inequalities in the prevalence of diabetes were present, and continued to persist over the period 2015–2023 suggest that past and current public health strategies have failed to promote and facilitate health among all socioeconomic groups in the Irish population. The higher risk of diabetes in persons of lower socio-economic status is thought to be the consequence of numerous potential factors including varied access to healthy foods and places to be physically active, health literacy and awareness, access to healthcare and other community services that affect risk factors, and because having more income enables one to seek and maintain a healthier diet [14, 16–19]. The higher prevalence of diabetes among socioeconomically disadvantaged groups could be the consequence of higher diabetes-related healthcare out-of-pocket costs [52]. For populations with lower income, diabetes management can be particularly difficult, specifically in receiving adequate support for medications, supplies, and care [53]. In Ireland, migration has contributed to the changing socioeconomic profile and diversification of the Irish population, which could further explain the persistence of these socioeconomic disparities [25].

Similarly, the higher diabetes prevalence among socioeconomically disadvantaged groups likely translates to higher risk of diabetes-related complications [54–57] which can adversely affect physical and mental health, social relationships, employment and further impede the socioeconomic and psychosocial circumstances of individuals living with diabetes [58, 59]. Failing to deal with inequalities in diabetes prevalence could further widen health disparities among the most vulnerable groups within the population [60]. To tackle inequalities, population-level interventions promoting healthy lifestyles e.g. nutritious diet, physical activity and targeted interventions to the needs of socioeconomically disadvantaged groups and individuals e.g. affordable housing, access to healthy foods, are required [61, 62]. To date, few interventions to prevent diabetes in Ireland have focused on socioeconomic inequalities [63].

We acknowledge several limitations in this study. In our analysis we were limited by the number of years and sample size to fully understand the nature of diabetes prevalence trends in Ireland and to detect changes in various population subgroups. Additionally, the socioeconomic indicators used in our study were limited to the data captured in the survey data. Education does not capture many aspects of wealth and socioeconomic status and is inversely associated with age, and its relationship to SES is likely changing over time. To overcome some of these challenges, we included age and sex interaction terms in the estimations. In addition, diabetes prevalence used in our analysis is based on self-reported data from survey respondents, and does not allow to differentiate by diabetes type. However, given that type 2 diabetes accounts for 90% to 95% of all diabetes cases [4], the likely dominance of type 2 diabetes is shaping patterns by SES and by age in this analysis. Although, historically it has been suggested that there was no association between deprivation and the prevalence (or incidence) of type 1 diabetes, there is new evidence to suggest that there is a gradient but it is much shallower than for type 2 diabetes [64]. As noted above, the surveys were not conducted in 2020 due to the COVID-19 pandemic. Therefore, we are not capturing diabetes prevalence and other socioeconomic information across a continued period from 2015 to 2023 The method of measuring and capturing area-based deprivation changed after 2015 [65] (from using the Small Area Health Research Unit index to using the HP index), therefore the deprivation measure used may vary relative to the other survey years. Additionally, no deprivation data was available in 2022, which limited our deprivation analyses over a shorter period 2016–2019, relative to education. Finally, our analysis did not account for diabetes-related risk factors and their changes over time, as these data were not consistently captured in the survey data over the period of our analysis.

Many of the abovementioned limitations could potentially be overcome, if the analysis were conducted using data from a national diabetes registry. Currently, there is no diabetes registry in Ireland, making it difficult to pinpoint Ireland’s performance in diabetes prevention and management, relative to other countries [66]. It is feasible to suggest that the likely dominance of type 2 diabetes is shaping patterns by SES and by age in this analysis. As type 1 diabetes is typically diagnosed at a younger age than type 2 diabetes, we would expect the opposite for our analysed population sample. Similarly, diabetes registry data could potentially help identify and inform clinicians and decision makers about which socioeconomic groups could further benefit from tailored and targeted diabetes programmes, to address the persisting inequalities in Ireland.

## Conclusion

Our study identified the persistence of significant differences in diabetes prevalence across socioeconomic status groups. The prevalence of diabetes in Ireland did not appear to increase over time, and relative inequalities in diabetes prevalence across socioeconomic groups were high and persisted over time. As the Irish population continues to age, this study highlights the need to consider potential effects of diabetes across the lowest socioeconomic status groups when implementing equity-oriented diabetes prevention and management programmes.

## Electronic supplementary material

Below is the link to the electronic supplementary material.

Supplementary Material 1

## Data Availability

The data that support the findings of this study are available from the Department of Health but restrictions apply to the availability of these data, which were used under license for the current study, and so are not publicly available. Data are however available from the authors upon reasonable request and with permission of the Department of Health.
