# Supplementary material for: Socioeconomic disparities in diabetes prevalence among the population in Ireland
Source: BMC Public Health. 2025 Jul 2;25:2206. doi: 10.1186/s12889-025-23022-6 (PMC12220495; doi:10.1186/s12889-025-23022-6)
Supplement: Supplementary file 1 — Supplementary Material 1 [file 12889_2025_23022_MOESM1_ESM.docx]

**Supplementary Information**

| **Year** | **2015** | **95% CI** | **2016** | **95% CI** | **2017** | **95% CI** | **2018** | **95% CI** | **2019** | **95% CI** | **2021** | **95% CI** | **2022** | **95% CI** | **2023** | **95% CI** | **Total** | **95% CI** |
| --- | --- | --- | --- | --- | --- | --- | --- | --- | --- | --- | --- | --- | --- | --- | --- | --- | --- | --- |
| **Total** | 4.0% | 3.7%,4.3% | 4.3% | 4%,4.6% | 4.2% | 3.9%,4.5% | 4.2% | 3.9%,4.5% | 4.4% | 4.1%,4.7% | 3.6% | 3.3%,3.9% | 4.8% | 4.5%,5.1% | 4.5% | 4.2%,4.8% | 4.2% | 3.9%,4.5% |
| **Sex** |  |  |  |  |  |  |  |  |  |  |  |  |  |  |  |  |  |  |
| **Male %** | 4.7% | 4.2%,5.2% | 5.3% | 4.8%,5.8% | 4.9% | 4.4%,5.4% | 4.9% | 4.4%,5.4% | 5.5% | 4.9%,6.0% | 4.1% | 3.5%,4.6% | 6.1% | 5.6%,6.6% | 4.4% | 3.9%,4.9% | 5.0% | 4.5%,5.5% |
| **Female %** | 3.3% | 2.9%,3.7% | 3.4% | 3.0%,3.8% | 3.5% | 3.1%,3.9% | 3.6% | 3.2%,3.9% | 3.3% | 2.9%,3.7% | 3.0% | 2.6%,3.4% | 3.5% | 3.1%,3.9% | 4.6% | 4.2%,4.9% | 3.5% | 3.1%,3.9% |
| **Age group** |  |  |  |  |  |  |  |  |  |  |  |  |  |  |  |  |  |  |
| **<40** | 0.8% | 0.5%,1.1% | 1.2% | 0.9%,1.5% | 1.3% | 1.0%,1.6% | 1.0% | 0.7%,1.3% | 0.4% | 0.1%,0.7% | 0.6% | 0.3%,0.9% | 1.0% | 0.7%,1.3% | 1.3% | 1.0%,1.6% | 1.0% | 0.7%,1.3% |
| **40-64** | 4.3% | 3.9%,4.6% | 4.1% | 3.8%, 4.4% | 4.2% | 3.9%,4.5% | 4.4% | 4.1%,4.7% | 4.9% | 4.6%,5.2% | 3.90% | 3.6%,4.2% | 5.1% | 4.7%,5.4% | 4.6% | 4.3%, 4.9% | 4.5% | 4.2%,4.8% |
| **65-74** | 9.4% | 8.8%,9.9% | 10.3% | 9.7%,10.9% | 10.5% | 9.9%,11.1% | 10.8% | 10.2%,11.4% | 12.3% | 11.7%,12.9% | 10.5% | 9.9%,11.1% | 10.6% | 10.0%,11.1% | 10.4% | 9.8%,10.9% | 10.6% | 10.0%,11.2% |
| **>75** | 15.4% | 13.3%,17.5% | 16.4% | 14.3%,18.5% | 12.4% | 10.3%,14.5% | 12.8% | 10.7%,14.9% | 12.7% | 10.6%,14.8% | 8.4% | 6.3%,10.5% | 15.6% | 13.5%,17.7% | 12.2% | 10.1%,14.3% | 13.3% | 11.2%,15.4% |
| **Education** |  |  |  |  |  |  |  |  |  |  |  |  |  |  |  |  |  |  |
| **Low** | 7.5% | 6.6%,8.4% | 8.1% | 7.2%,9.0% | 7.6% | 6.7%,8.5% | 8.1% | 7.2%,9.0% | 8.4% | 7.5%,9.3% | 6.5% | 5.6%,7.4% | 10.2% | 9.3%,11.1% | 8.8% | 7.9%,9.7% | 8.1% | 7.2%,9.0% |
| **Medium** | 2.6% | 2.3%,2.9% | 3.0% | 2.7%,3.3% | 2.9% | 2.6%,3.2% | 3.2% | 2.9%,3.5% | 2.9% | 2.6%,3.2% | 2.6% | 2.3%,2.9% | 2.8% | 2.5%,3.1% | 3.7% | 3.4%,4.0% | 2.9% | 2.6%,3.2% |
| **High** | 1.4% | 1.1%,1.7% | 1.2% | 0.9%,1.5% | 1.5% | 1.2%,1.8% | 1.5% | 1.2%,1.8% | 2.1% | 1.8%.2.4% | 1.7% | 1.4%,1.9% | 1.9% | 1.6%,2.2% | 2.2% | 1.9%,2.5% | 1.7% | 1.4%,2.8% |
| **Deprivation** |  |  |  |  |  |  |  |  |  |  |  |  |  |  |  |  |  |  |
| **1 - Most deprived** | - | - | 6.9% | 5.8%,8.0% | 6.0% | 4.9%,7.1% | 5.2% | 4.1%,6.3% | 5.7% | 4.6%,6.8% | - | - | - | - | - | - | 6.0% | 4.9%,7.1% |
| **2** | - | - | 4.4% | 3.6%,5.2% | 4.2% | 3.4%,5.0% | 4.7% | 3.9%,5.5% | 3.5% | 2.7%,4.3% | - | - | - | - | - | - | 4.2% | 3.4%,5.0% |
| **3** | - | - | 4.1% | 2.3%,5.9% | 3.6% | 1.8%,5.4% | 6.1% | 4.3%,7.9% | 5.2% | 3.4%,6.9% | - | - | - | - | - | - | 4.8% | 3.0%,6.6% |
| **4** | - | - | 3.3% | 1.6%,4.9% | 4.8% | 3.1%,6.5% | 2.3% | 0.6%,3.9% | 4.0% | 2.3%,5.7% | - | - | - | - | - | - | 3.6% | 1.9%,5.3% |
| **5 - Least deprived** | - | - | 2.3% | 1.5%,3.2% | 1.6% | 0.8%,2.5% | 2.2% | 1.4%,3.1% | 2.9% | 2.1%,3.8% | - | - | - | - | - | - | 2.2% | 1.4%,3.1% |

**Supplementary File 1**

**Table A1.** Diabetes prevalence by survey year for each SES indicator and individual demographics (2015-2023)

Note: Population sampling weights provided in the Healthy Ireland survey were applied in estimation. Data for area-based deprivation was limited to years 2016-2019 only.

**Supplementary File 2**

**Figure A1.** Self-reported diabetes prevalence as per Healthy Ireland survey period 2015 - 2023

**Figure A2.** Age-group variation in diabetes prevalence as per Healthy Ireland survey period 2015 - 2023

**Supplementary File 3**

**Table A2.** Adjusted Relative Risk ratios of self-reported diabetes by age-group (2015-2023)

|  | **RR** | **95% Confidence Interval** | **Significance level** |
| --- | --- | --- | --- |
|  | **Age-group** | | |
| **<40 years** | Ref | Ref | Ref |
| **40-64 years** | 4.43 | 3.85, 5.11 | *** |
| **>65 years** | 9.11 | 7.81, 10.64 | *** |
| **>75 years** | 10.9 | 9.34, 12.92 | *** |
| **N** | 59,768 | | |

Note: Separate logistic regression models were conducted for age-group adjusted for sex, survey year. Age-group <40 years was the reference category.

**Supplementary File 4**

**Table A3.** Relative index of inequality (RII) by SES by survey year and full survey period (2015 – 2023)

| **Year** | **2015** | **2016** | **2017** | **2018** | **2019** | **2021** | **2022** | **2023** | **2015 - 2023** |
| --- | --- | --- | --- | --- | --- | --- | --- | --- | --- |
|  | **Education** | | | | | | | | |
| **RII** | 3.44  (2.19, 5.37) | 3.68  (2.41, 5.61) | 3.22  (2.09, 4.97) | 4.36  (2.84, 6.69) | 3.95  (2.56, 6.08) | 2.30  (1.39, 3.81) | 3.46  (2.22, 5.41) | 3.67  (3.36, 5.72) | 3.90  (3.31,4.59) |
| **p-value** | <0.01 | <0.01 | <0.01 | <0.01 | <0.01 | <0.01 | <0.01 | <0.01 | <0.01 |
|  | **Deprivation** | | | | | | | | |
| **RII** | - | 2.67  (1.79, 3.98) | 1.58  (1.06, 2.36) | 2.09  (1.42, 3.09) | 1.54  (1.05, 2.28) | - | - | - | 3.65  (2.48,5.37) |
| **p-value** | - | <0.01 | <0.01 | <0.01 | <0.01 | - | - | - | <0.01 |

Note: 95% Confidence Intervals reported in parentheses. All RII estimates were adjusted for sex and age. Area-based deprivation data were available for 2016 – 2019 only.

**Supplementary File 5**

**Table A4**. Summary of adjusted relative risk ratios for testing age-group and socioeconomic differences in diabetes prevalence over time (trends)

|  | **Diabetes - Yes** | | **Diabetes - No** |
| --- | --- | --- | --- |
|  | **RR** | **p-value** |  |
| Age-group | 1.00  (0.98, 1.01) | 0.896 | Reference |
| Education | 1.01  (0.99, 1.02) | 0.304 | Reference |
| Deprivation | 0.99  (0.95, 1.05) | 0.950 | Reference |

Note: Reported are estimated adjusted relative risk ratios from a logistic regression adjusted for sex*age (interaction), survey year for education and area-based deprivation. Age-group estimation adjusted for sex and survey year. A (non)significant RR indicates (non) statistically significant differences in diabetes prevalence by age-group and SES over the survey period. 95% Confidence Intervals reported in parentheses.

**Supplementary File 6**

**Table A5.** Summary of the full combined sample of individuals used in analysis by demographic and socioeconomic variables (2015-2023)

|  |  |
| --- | --- |
| **Variable** | Total |
| n (%) | 59933 |
| **Sex, n (%)** |  |
| Male | 29376 |
| Female | 30548 |
| **Age group, n (%)** |  |
| <40 | 25136 |
| 40 – 64 | 24333 |
| 65 - 74 | 6325 |
| > 75 | 4137 |
| **Education level, n (%)** |  |
| Low: </primary, lower secondary | 18796 |
| Medium: secondary, post-secondary, non-tertiary | 24861 |
| High: 3rd level, BA MA PhD | 16207 |
| **Deprivation, n (%)** |  |
| 1 – Most deprived | 6486 |
| 2 | 6251 |
| 3 | 6139 |
| 4 | 6022 |
| 5 – Least deprived | 5094 |
| **Year, n (%)** |  |
| 2015 | 7539 |
| 2016 | 7498 |
| 2017 | 7487 |
| 2018 | 7701 |
| 2019 | 7382 |
| 2021 | 7454 |
| 2022 | 7455 |
| 2023 | 7411 |

Note: full sample includes all individuals with and without self-reported diabetes

**Supplementary File 7**

**Table A6.** Sensitivity analysis limited to individuals aged >30 years: Adjusted Relative Risk ratios of self-reported diabetes by education (2015 – 2023)

|  | **RR** | **95% Confidence Interval** | |
| --- | --- | --- | --- |
|  | **Education level** | | |
| **Low** | 2.91 | 2.53 | 3.35 |
| **Medium** | 1.62 | 1.40 | 1.80 |
| **High** | Ref | Ref | Ref |
| **N** | 51,313 | | |
